# Effect of Substrate Temperature on the Structural, Optical and Electrical Properties of DC Magnetron Sputtered VO_2_ Thin Films

**DOI:** 10.3390/ma15217849

**Published:** 2022-11-07

**Authors:** Chunzi Zhang, Ozan Gunes, Shi-Jie Wen, Qiaoqin Yang, Safa Kasap

**Affiliations:** 1 Department of Electrical and Computer Engineering, University of Saskatchewan, Saskatoon, SK S7N 5A9, Canada; 2 Cisco Systems Inc., 170 West Tasman Drive, San Jose, CA 95134, USA; 3 Department of Mechanical Engineering, University of Saskatchewan, Saskatoon, SK S7N 5A9, Canada

**Keywords:** vanadium dioxide thin films, substrate temperature, magnetron sputtering, optical transmittance, electrical conductivity, hysteresis, sharpness, phase transition

## Abstract

This study focuses on the effect of the substrate temperature (*T_S_*) on the quality of VO_2_ thin films prepared by DC magnetron sputtering. *T_S_* was varied from 350 to 600 °C and the effects on the surface morphology, microstructure, optical and electrical properties of the films were investigated. The results show that *T_S_* below 500 °C favors the growth of V_2_O_5_ phase, whereas higher *T_S_* (≥500 °C) facilitates the formation of the VO_2_ phase. Optical characterization of the as-prepared VO_2_ films displayed a reduced optical transmittance ($\tilde{T}$) across the near-infrared region (NIR), reduced phase transition temperature (*T_t_*), and broadened hysteresis width (Δ*H*) through the phase transition region. In addition, a decline of the luminous modulation $\left(\Delta {\tilde{T}}_{\mathrm{lum}}\right)$ and solar modulation ($\Delta {\tilde{T}}_{\mathrm{sol}}$) efficiencies of the as-prepared films have been determined. Furthermore, compared with the high-quality films reported previously, the electrical conductivity (*σ*) as a function of temperature (*T*) reveals reduced conductivity contrast (Δ*σ*) between the insulating and metallic phases of the VO_2_ films, which was of the order of 2. These outcomes indicated the presence of defects and unrelaxed lattice strain in the films. Further, the comparison of present results with those in the literature from similar works show that the preparation of high-quality films at *T_S_* lower than 650 °C presents significant challenges.

## 1. Introduction

Vanadium dioxide (VO_2_) has been widely studied for applications such as optical switches, sensors, smart windows, and optical memory devices [1,2,3,4]. Currently, potential applications can also be found in neuromorphic computing materials and metamaterials [5,6,7,8]. The primary reason for its importance in electronics, photonics and optical switching applications is the fast metal-to-insulator phase transition (MIT) between the semiconductor and metallic phases. During the MIT, the VO_2_ crystal undergoes an abrupt phase transformation from high temperature tetragonal phase to a low temperature monoclinic phase at around 68 °C [9]. Up to now, a variety of deposition methods (i.e., magnetron sputtering, pulsed laser deposition, chemical vapor deposition, electron beam evaporation etc.) have been employed to prepare VO_2_ thin films [10,11,12,13,14,15]. Regardless of the method, high growth temperatures (450–800 °C) are employed to assure its stoichiometry [16,17,18,19], which is crucial in the synthesis of VO_2_ thin films that display a sharp phase transition. However, instrumentation that delivers high substrate temperatures (*T_S_*) is costly and not industry-friendly [20]. For this reason, there have been numerous efforts to bring the *T_S_* down to more practical temperatures [21,22,23,24,25,26,27]. A few studies have already reported the fabrication of the VO_2_ thin films at low *T_S_* (down to around 300 °C) by applying a buffer layer such as ZnO, AZO, TiO_2_, SnO_2_ or VO [21,22,23]. Other pronounced efforts typically include substrate biasing, applying high power pulse, and post-annealing [24,25,26,27]. In order to reduce the *T_S_*, a clear understanding of the effect of the synthesis (substrate) temperature on the stoichiometry, structure and phase transition properties is critical. Some previous works have already revealed the effects of *T_S_* on the microstructure, stress state, and transition temperatures of VO_2_ thin films [16,28,29,30,31]. Nevertheless, these works are not comprehensive and do not necessarily signify a clear relation of the underlying mechanism related to physical changes due to the *T_S_*.

In our earlier works [17,32], we have reported the preparation of stoichiometric, near-epitaxial VO_2_ thin films at *T_S_* of 650 °C by DC reactive magnetron sputtering, where the films showed highly discernable optical and electrical phase transition properties (i.e., high optical transmittance ($\Delta \tilde{T}$) and electrical conductivity (Δ*σ*) contrasts, sharp transitions and near-zero optical transmission in the metallic phase). We have also investigated the effect of substrate biasing on the quality of VO_2_ thin films [33]. In the present work, we have undertaken the deposition of VO_2_ films at *T_S_* varying from 350 to 600 °C and examined the effects on the microstructure, surface morphology, roughness, optical and electrical phase transition properties. We compared these results with the films deposited at 650 °C in our previous works [17,32]. In addition, we extracted and analyzed the $\Delta \tilde{T}$ along with the luminous $\left(\Delta {\tilde{T}}_{\mathrm{lum}}\right)$ and solar ($\Delta {\tilde{T}}_{\mathrm{sol}}$) optical modulation efficiencies. Furthermore, we estimated the electrical bandgap of the films and compared the results with those of high-quality VO_2_ thin films prepared at 650 °C reported previously [32]. Additionally, we compared our results with other works that studied the effect of *T_S_* on the properties of VO_2_ thin films.

## 2. Materials and Methods

Single crystal p-Si (100) and r-cut sapphire were used as substrates for the thin film preparation. Prior to the deposition, the substrates were ultrasonically cleaned in ethanol and dried in air. After the cleaning process, the substrates were placed in a sputtering chamber. DC reactive magnetron sputtering was utilized to prepare VO_2_ thin films. For this purpose, a high-purity vanadium target (99.95%, Plasmionique, Inc., Varennes, QC, Canada) was used. During every deposition, stable argon (Ar) and oxygen (O_2_) flow with constant rates of 100 sccm and 1.3 sccm were maintained, respectively. The selected Ar and O_2_ flow rates facilitate the growth of pure VO_2_ films using the above-mentioned deposition process which has been also reported in Ref. [17]. All depositions were performed under a constant pressure of 1.3 Pa for a duration of 2 h. The thin films were prepared at six different *T_S_* values. The deposition conditions for each *T_S_* are summarized in Table 1.

After deposition, the structural properties of the as-prepared thin films were examined at room temperature using Raman spectroscopy (Renishaw Invia Reflex Raman Microscope, Wharton-under-Edge, UK), scanning electron microscopy (Hitachi HT-7700 SEM, Tokyo, Japan), and X-ray diffractometry (Rigaku Ultima IV XRD, Tokyo, Japan). The average crystalline grain size was estimated using ImageJ (a software program developed by NIH, Bethesda, MD, USA [34]) on SEM images. Surface profilometry was used to examine the surface roughness of the films using a profilometer (Zygo OMP-0592, Middlefield, CT, USA). The optical transmittance spectrum was measured using a Lambda 900 UV-Vis-NIR Perkin-Elmer spectrophotometer (Waltham, MA, USA) in the wavelength (*λ*) range of 300–2500 nm. During the optical measurements, VO_2_-film/substrate sample was mounted on a purpose-built sample heating platform connected to a DC power supply (similar to the one in Ref. [32]). An Omega ^®^ CN-16Pt-330 PID temperature controller (La Chaux-de-Fonds, Switzerland) was used to control the sample holder temperature and monitor the sample temperature during the heating and cooling processes. The temperature (*T*) dependence of the optical transmittance ($\tilde{T}$) measurements were conducted at a heating rate of ~0.5 °C/min. The *T*-dependence of the conductivity (*σ*) of the as-prepared films on sapphire substrates were carried out by depositing gold (Au) contacts at room temperature with the size of (1 mm × 2 mm) using a radio frequency (RF) sputtering system. These measurements were carried out in a cryostat where the samples were placed on a copper (Cu) block which was connected to a DC power supply. The supply was used to heat the block. The temperature of the samples was measured by the use of a thermocouple which was mounted in close proximity to the sample. During these measurements, the heating of the sample was specifically kept at a low rate (~0.5 °C/min) to prevent a lag between the temperature reading and the actual temperature of the sample.

## 3. Results

### 3.1. Microstructural Characterization by Raman Spectroscopy and XRD

**Figure 1:**
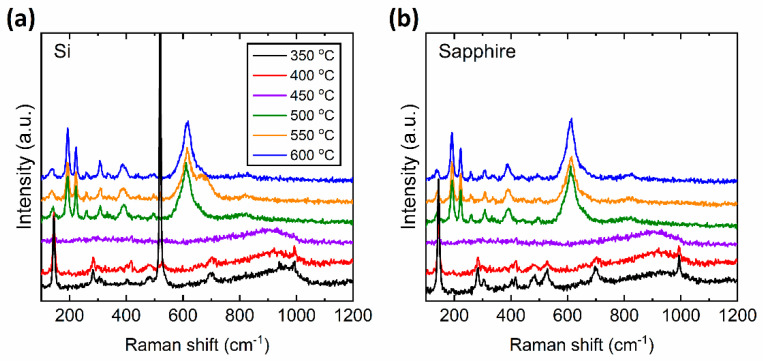
Raman spectra of VO_2_ and V_2_O_5_ thin films prepared at various substrate temperatures (*T_S_*) on (**a**) Si and (**b**) sapphire substrates. Note that the vertical axes are in arbitrary units (a.u.), so the relative magnitudes of the spectra cannot be compared.

**Figure 2:**
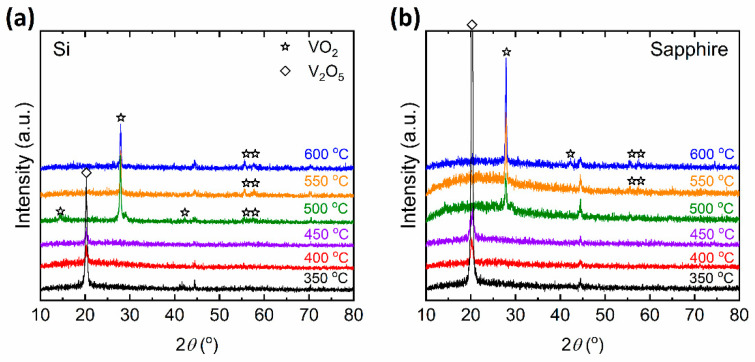
XRD spectra of VO_2_ and V_2_O_5_ thin films prepared at various substrate temperatures (*T_S_*) on (**a**) Si and (**b**) sapphire substrates. Note that the vertical axes are in arbitrary units (a.u.).

The microstructural characterization of the vanadium oxide thin films prepared at different *T_S_* was carried out using Raman spectroscopy and x-ray diffraction (XRD) for which the results can be found in Figure 1 and Figure 2, respectively. These figures show that the pure monoclinic VO_2_ phase is achieved only for samples deposited at *T_S_* = 500 °C and higher temperatures. In Figure 1, the Raman peaks at 144, 193, 223, 260, 308, 334, 389, 497 and 613 cm^−1^ represent the pure monoclinic phase of VO_2_ [15]. In Figure 2, the diffraction peaks at 2*θ* = 28°, 56°, and 58° correspond to the (011), (220), and (022) of pure monoclinic phase of VO_2_ [35]. For *T_S_* = 450 °C, the Raman peaks of pure VO_2_ are not observed in Figure 1 and a small diffraction peak at 2*θ* = 20° appears in Figure 2, which corresponds to the (001) peak of V_2_O_5_. As the *T_S_* is further decreased down to 350 °C, the (001) peak of V_2_O_5_ dominates the XRD spectrum as can be seen in Figure 2a,b. The Raman peaks at 145, 195, 284, 303, 405, 483, 528, 701, and 996 cm^−1^ in Figure 1a,b, signify the presence of V_2_O_5_ phase to be the dominant in the thin film [15] below a *T_S_* of 450 °C. It can be seen that both Raman and XRD peaks of V_2_O_5_ become the strongest when the *T_S_* reaches 350 °C. It is worth mentioning that the sharp Raman peak around 520 cm^−1^ for the film deposited *T_S_* = 350 °C on a Si substrate arises from the Si substrate itself and is absent in films deposited on sapphire substrates.

### 3.2. Surface Morphology and Roughness Characterization by SEM and Profilometry

**Figure 3:**
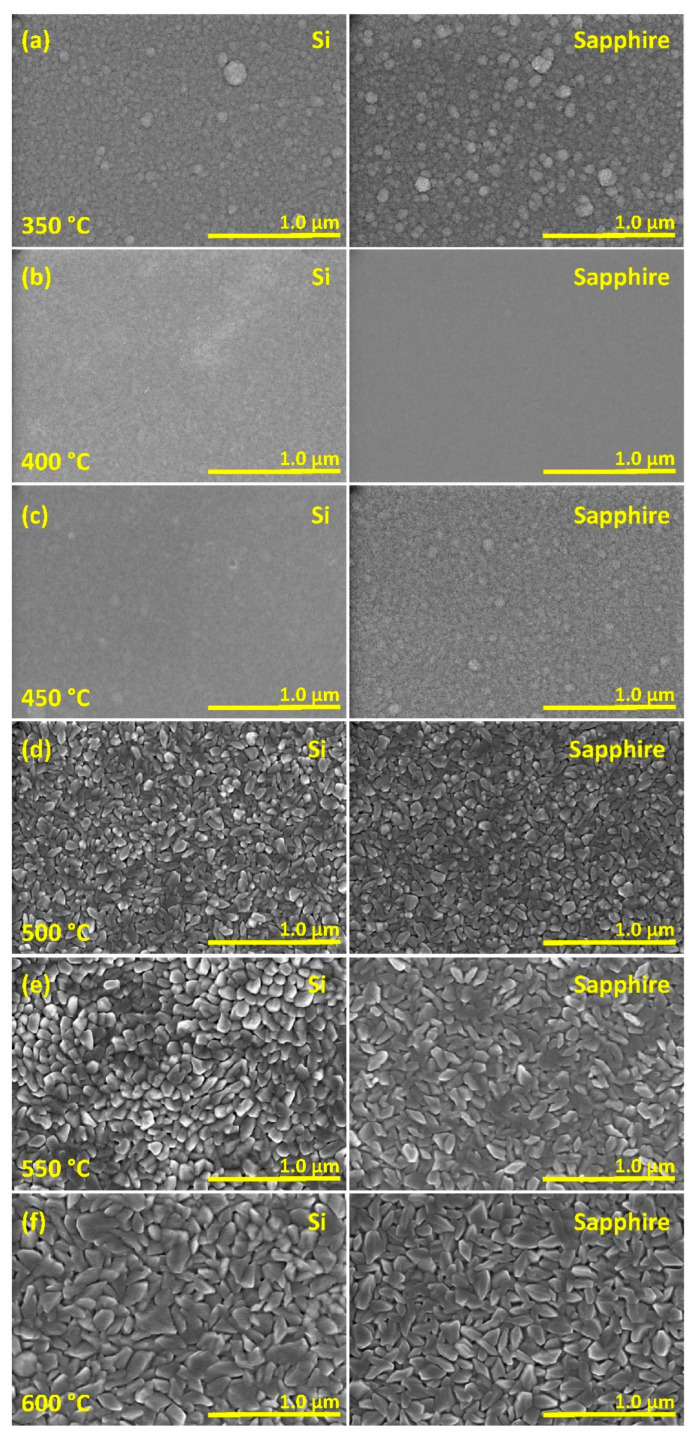
Surface images of VO_2_ and V_2_O_5_ thin films prepared at various substrate temperatures (*T_S_*) (**a**) 350 °C, (**b**) 400 °C, (**c**) 450 °C, (**d**) 500 °C, (**e**) 550 °C, and (**f**) 600 °C obtained by SEM.

**Figure 4:**
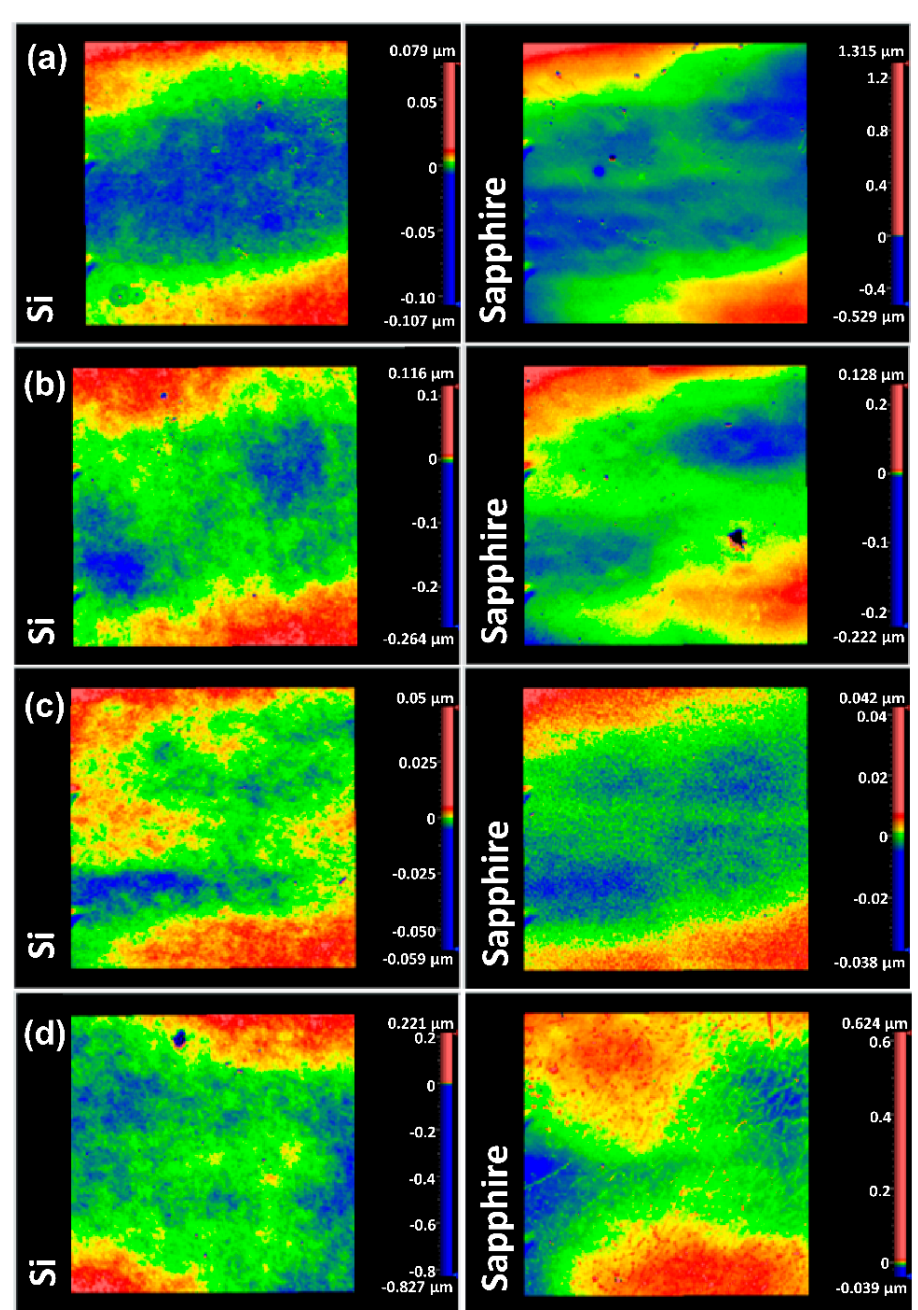
Surface profile images of VO_2_ thin films deposited on Si and sapphire substrates at (**a**) 500 °C, (**b**) 550 °C, (**c**) 600 °C and (**d**) 650 °C [17]. The roughness of the surface is indicated by color mapping, where the maximum (peak), median and minimum (depth) points on the surface are indicated in red, green and blue, respectively. The scale bars are used as a measure of the peaks and depths on the surface of the films.

**Figure 5:**
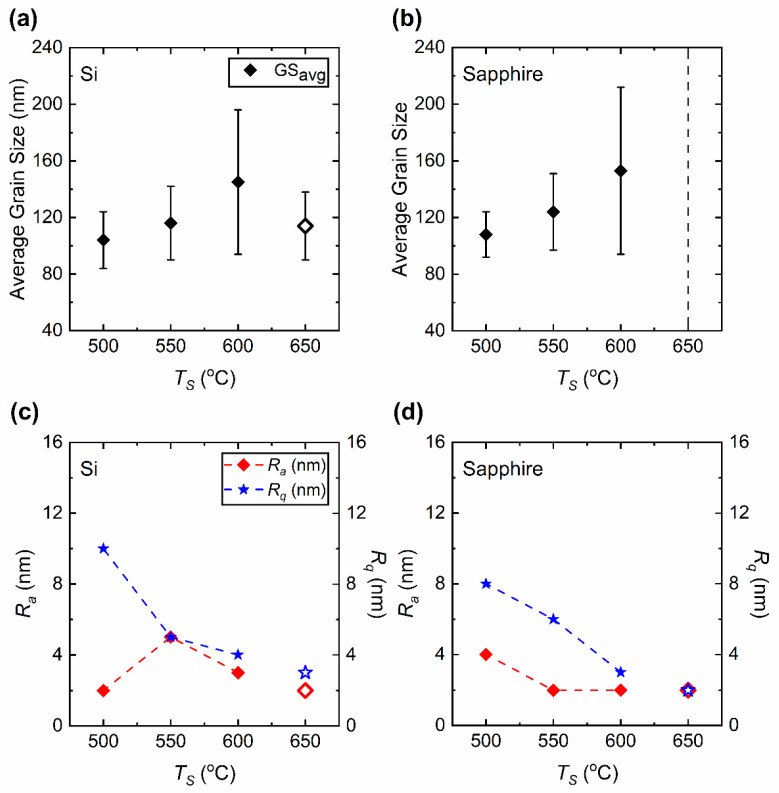
Average grain size (GS_avg_) of VO_2_ thin films prepared on (**a**) Si and (**b**) sapphire substrates. In (**b**) the dashed vertical line is an asymptote that indicates the near-epitaxial almost flat surface of the film prepared at 650 °C [17]. (**c**,**d**) present the average (*R_a_*) and root-mean-square (*R_q_*) roughness of films prepared on Si and sapphire substrates, respectively. The dashed lines between the data points are guides to the eye. The data points shown in hollow symbols belong to the films prepared at 650 °C (prepared as described in Ref. [17] but not reported therein).

Figure 3 presents the SEM surface images of the as-prepared VO_2_ thin films. It can be observed that the *T_S_* affects the surface morphology of the thin films. As observed from (a) to (b) to (c) the films show a misoriented crystalline structure with grain sizes ranging between around 100 and 150 nm. The substrate temperatures of 450 and 400 °C produce V_2_O_5_ nanostructured films with smooth surfaces. When the *T_S_* is lowered to 350 °C the V_2_O_5_ grains become clearly discernable in the films. Surface profilometry shows that *T_S_* also affects the surface roughness of VO_2_ films. Figure 4 shows the surface profile images of the VO_2_ films deposited at various *T_S_*-values. These images are taken at a surface area of approximately 0.6 × 0.6 mm^2^ of each film. Roughness measurements show that the root-mean-square (*R_q_*) roughness of films on both Si and sapphire substrates increases as *T_S_* is lowered. The maximum *R_q_* measured was obtained for VO_2_ films deposited at 500 °C, with 10 nm for films on Si and 8 nm for films on sapphire substrates. The minimum *R_q_* is measured for films deposited at 600 °C as 4 nm on Si and 3 nm on sapphire substrates. Yet, these films show increased roughness compared to the films prepared at 650 °C [17]. The *R_q_* is measured as 3 nm on Si and 2 nm on sapphire substrates for films prepared at 650 °C. These, in fact, manifest properties associated with high-quality VO_2_ films as investigated previously [36]. Nevertheless, the average roughness (*R_a_*) does not exhibit a strong variation with respect to *T_S_*, in contrast to the clear trend exhibited by *R_q_*. Table 2 and Figure 5 present the average grain size (GS_avg_), *R_a_* and *R_q_* of the VO_2_ thin films prepared on Si and sapphire substrates. The grain size in the films on sapphire substrates decreases from 153 nm to 108 nm as *T_S_* is lowered from 600 to 500 °C. Indeed, near-epitaxial growth on sapphire films has been reported in the literature [37]. On the other hand, the grain size of the films prepared on Si substrates, within experimental uncertainties, does not exhibit any compelling dependence on *T_S_*, even though there is seemingly a maximum average grain size at *T_S_* = 600 °C. Films on Si substrates prepared at 600 °C show grains with largely varying size (large standard deviation) and orientation. Even though the grain size is reduced for films prepared at *T_S_* = 550 and 500 °C the misorientation of grains is still evident. The thickness of the VO_2_ films prepared at *T_S_* = 600, 550 and 500 °C are measured by using cross-section SEM images as 210 ± 8, 230 ± 14, and 289 ± 15 nm, respectively.

### 3.3. Optical Characterization by Spectrophotometry

**Figure 6:**
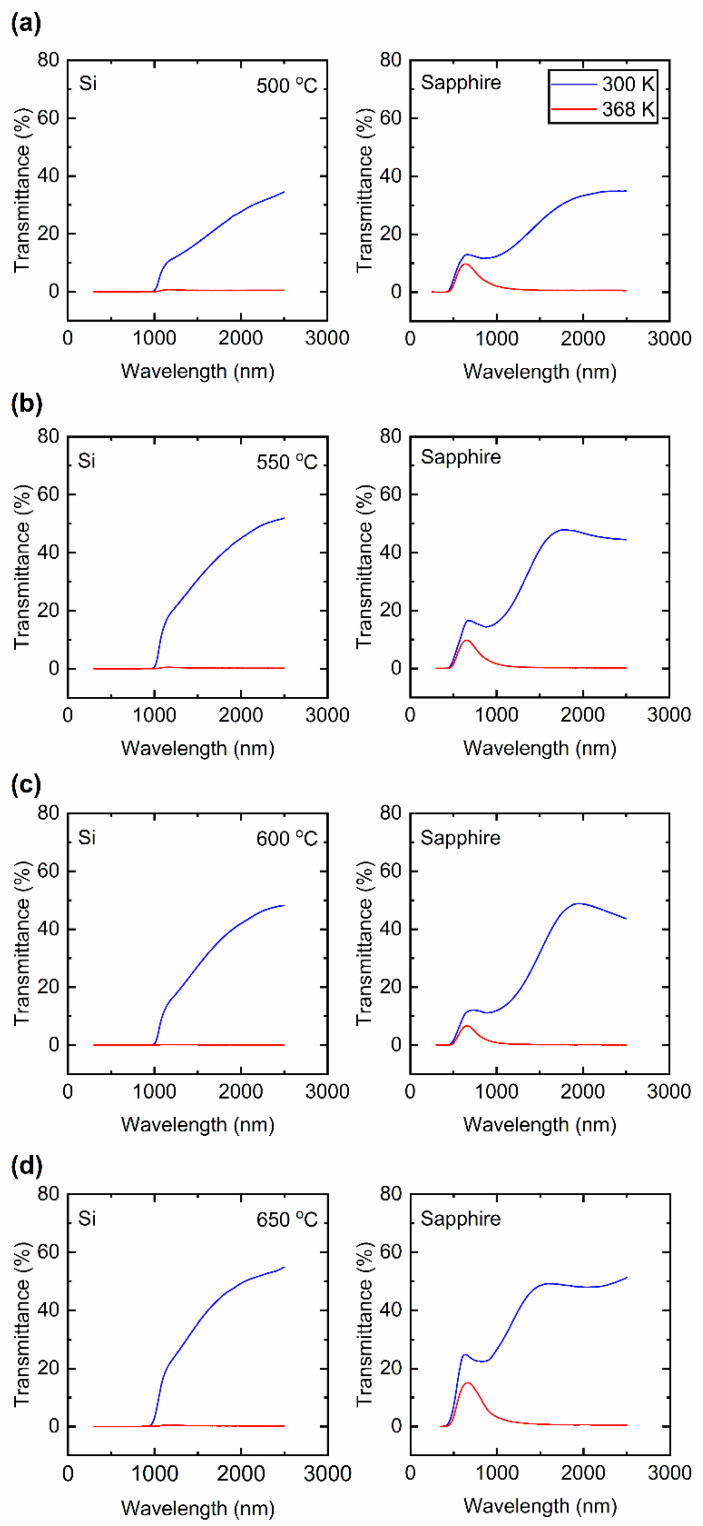
Optical transmittance ($\tilde{T}$) spectra of VO_2_ thin films deposited at (**a**) 500 °C, (**b**) 550 °C, and (**c**) 600 °C and (**d**) 650 °C [17] on Si and sapphire substrates at 300 K (insulating phase) and 368 K (metallic phase).

To understand the effect of *T_S_* on the structural VO_2_ phase transition properties, the optical transmission ($\tilde{T}$) spectrum of VO_2_ thin films was measured at 300 K (27 °C) (in the insulating phase) and at 368 K (95 °C) (in the metallic phase) in a wavelength range between 300 and 2500 nm. Figure 6 presents the $\tilde{T}$ spectra of these VO_2_ thin films. In practical terms, no notable difference can be found in the trend of the $\tilde{T}$ spectra for samples prepared at *T_S_* = 600 and 550 °C. In Figure 6a, for the film prepared at *T_S_* = 500 °C, ${\tilde{T}}_{2500\mathrm{nm}}$ is observed to be the lowest among other examined films, which is around 34% in the insulating phase. In Figure 6b,c the $\tilde{T}$ of films deposited at *T_S_* = 550 and 600 °C on sapphire are observed to be very close. The drop of the $\tilde{T}$ for films prepared at 500 °C can be related to the increased defects and the inferior crystal quality of VO_2_ thin films, which can be clearly observed in Figure 3d. At 368 K, all the VO_2_ films on both Si and sapphire substrates transition into a near-zero transmittance in the metallic phase. Table 3 presents the details of $\tilde{T}$ values at 300 K and 368 K along with the optical transmittance contrast ($\Delta \tilde{T}$) of the as-prepared VO_2_ films obtained at NIR wavelengths of 1500, 2000 and 2500 nm. $\Delta \tilde{T}$ is defined as the difference in the optical transmittance of the insulating and metallic phases.

**Figure 7:**
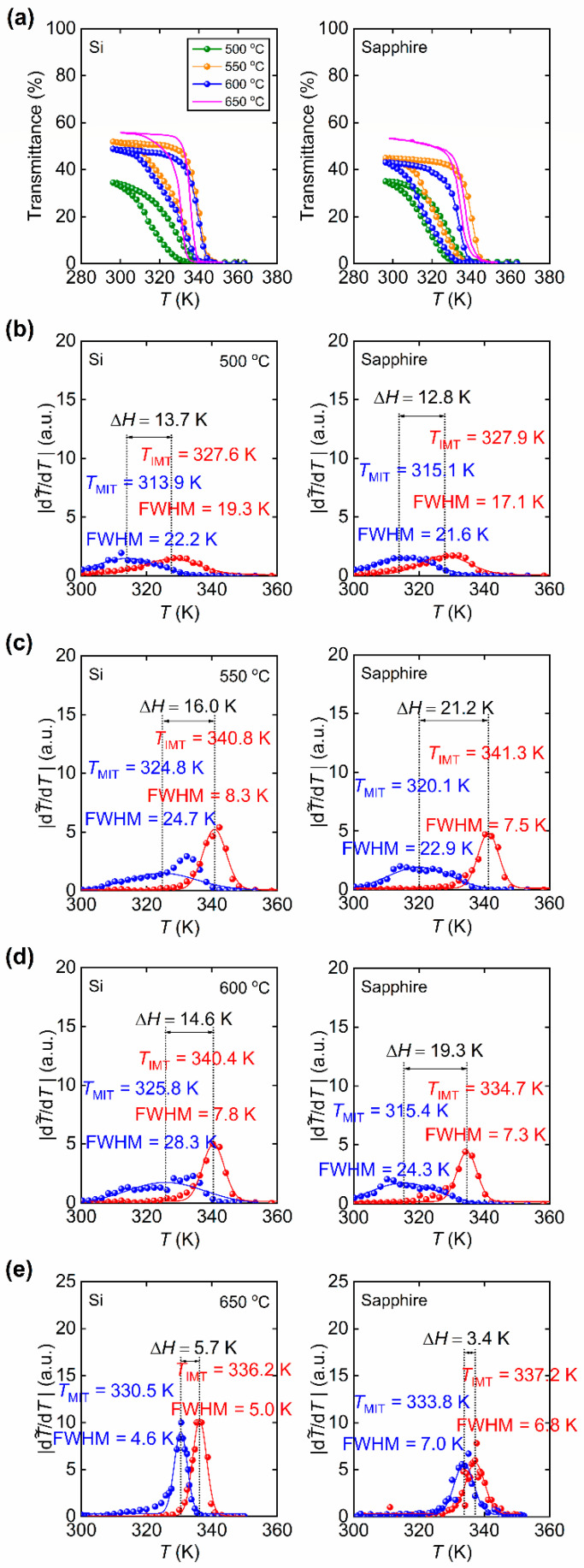
(**a**) Temperature dependence of optical transmittance ($\tilde{T}$) obtained at a light wavelength of *λ* = 2500 nm for VO_2_ thin films deposited at 500, 550, 600 and 650 °C [17]. The phase transition characteristics for heating and cooling cycles for films prepared at (**b**) 500 °C, (**c**) 550 °C, (**d**) 600 °C, and (**e**) 650 °C [17] on Si and sapphire substrates at 300 K (insulating phase) and 368 K (metallic phase).

Figure 7a gives the temperature dependence of ${\tilde{T}}_{2500\mathrm{nm}}$ of the VO_2_ thin films prepared at various *T_S_*-values. Figure 7b–e show the $d\tilde{T}/dT$ vs. *T* plots which give the critical temperatures (*T*_IMT_ and *T*_MIT_) and sharpness (full width at half maximum, FWHM) of the IM and MI phase transitions. It is important to emphasize that the narrow and sharp hysteresis of IM and MI transitions in VO_2_ films deposited at 650 °C on sapphire substrate in Ref. [17] is not observed in the thin films deposited at *T_S_* = 600 °C and below. The hysteresis width (Δ*H*) of the films deposited on both Si and sapphire substrates become broader as the *T_S_* is lowered. The broadening appears to be about 14–16 K for films on Si and 13–21 K for films on sapphire.

**Figure 8:**
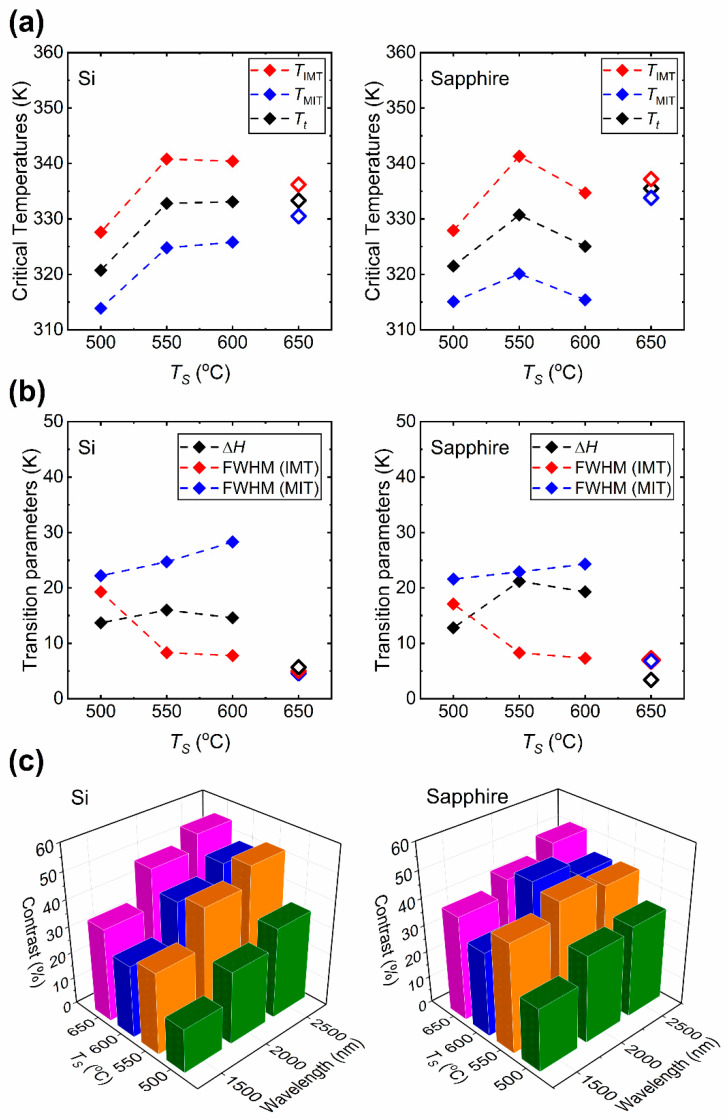
(**a**) Critical temperatures (*T*_IMT_, *T*_MIT_ and *T_t_*) of the VO_2_ films on Si and sapphire prepared at various substrate temperatures (*T_S_*) obtained at a light wavelength of *λ* = 2500 nm. (**b**) Hysteresis (Δ*H*) and sharpness (FWHM) of the Gaussian curves obtained from $d\tilde{T}/dT$ vs. *T* plots in Figure 7. In (**a**) and (**b**) the data points for films prepared at 650 °C [17] are shown in hollow symbols. (**c**) Comparison of optical transmittance contrast, $\Delta \tilde{T}=\tilde{T}(300\;K)-\tilde{T}\left(368\;K\right)$, obtained at NIR wavelengths *λ* = 1500 nm, 2000 nm and 2500 nm for films prepared at *T_S_* = 500 (olive), 550 (orange), 600 (navy) and 650 [17] (magenta) °C.

Figure 8 presents the comparison of phase transition properties obtained at *λ* = 2500 nm. In Figure 8a the insulator-to-metal (*T*_IMT_), metal-to-insulator (*T*_MIT_) and the phase transition (*T_t_*) temperatures are presented. The *T_t_* is calculated as the mean of the *T*_IMT_ and *T*_MIT_ and is given in Equation (1) below:

(1) $$T_{t}=\frac{T_{\mathrm{IMT}}+T_{\mathrm{MIT}}}{2}$$

By definition, *T_t_* depends on the hysteresis width (Δ*H*). It is observed that the critical temperatures on silicon substrates are relatively the same as *T_S_* is lowered from 650 [17] to 550 °C, but eventually decrease sharply for films prepared at 500 °C. For films on Si, as the *T_t_* stays approximately constant for *T_S_* = 650, 600 and 550 °C at about 340 K but falls to 320 K for *T_S_* = 500 °C. On the other hand, the *T_t_* of the films on sapphire appear to be lower compared to that for the film prepared at 650 °C in Ref. [17], displaying the lowest *T_t_* around 322 K at 500 °C. There is no clear dependence of *T_t_* on *T_S_* for films prepared on sapphire substrates. This change in *T_t_* indicates increased defects in the VO_2_ films. In addition, the change may be attributed to the spatial variation of local *T*_IMT_ and *T*_MIT_ due to increased number of coexisting metallic and insulating phases within the volume of the films as pointed out elsewhere [38]. Figure 8b presents the Δ*H* and the full width half maximum (FWHM) of the IM and MI transitions of the as-prepared VO_2_ thin films. The increasing FWHM can be interpreted as the degrading sharpness of the phase transitions. Figure 8c introduces the comparison of transmittance contrast ($\Delta \tilde{T}$) of the as-prepared films at *λ* = 1500, 2000, and 2500 nm which are commonly considered in NIR applications [39,40,41,42,43]. At lower *T_S_*, the Δ*H* becomes broader. The luminous (visible) and solar transmission characteristics of the as-prepared VO_2_ films deposited on sapphire substrates are also analyzed. The luminous transmittance (${\tilde{T}}_{\mathrm{lum}}$), solar transmittance (${\tilde{T}}_{\mathrm{sol}}$), the luminous modulation $\left(\Delta {\tilde{T}}_{\mathrm{lum}}\right)$ and solar modulation ($\Delta {\tilde{T}}_{\mathrm{sol}}$) efficiencies are commonly considered properties for smart window applications [44,45,46,47,48,49,50]. ${\tilde{T}}_{\mathrm{lum}}$ measures the amount of visible light passing through a semi-transparent material. ${\tilde{T}}_{\mathrm{sol}}$ is considered as an index that characterizes the transmission of sunlight within the visible and near-infrared spectrum. ${\tilde{T}}_{\mathrm{lum}}$ and ${\tilde{T}}_{\mathrm{sol}}$ can be determined from Equation (2) below:

(2) $${\tilde{T}}_{\mathrm{lum},\mathrm{sol}}=\frac{\int_{}^{}\varphi_{\mathrm{lum},\mathrm{sol}}(\lambda )\tilde{T}(\lambda )d\lambda }{\int_{}^{}\varphi_{\mathrm{lum},\mathrm{sol}}(\lambda )d\lambda }$$

${\tilde{T}}_{\mathrm{lum}}$ is obtained for a wavelength range between 380 and 780 nm and ${\tilde{T}}_{\mathrm{sol}}$ is obtained for wavelengths between 300 and 2500 nm. $\varphi_{\mathrm{lum}}(\lambda )$ is the spectral sensitivity of the light adapted human eye [51], $\varphi_{\mathrm{sol}}(\lambda )$ is the solar irradiance spectrum for air mass of 1.5 (37° sunlight above the horizon of earth) [52], and $\tilde{T}(\lambda )$ is acquired from the transmittance spectrum of the as-prepared films. $\Delta {\tilde{T}}_{\mathrm{lum}}$ and $\Delta {\tilde{T}}_{\mathrm{sol}}$ are calculated from the Equation (3) given below:

(3) $$\Delta {\tilde{T}}_{\mathrm{lum},\;\mathrm{sol}}={\tilde{T}}_{\mathrm{lum},\;\mathrm{sol}}(300\;K)-{\tilde{T}}_{\mathrm{lum},\;\mathrm{sol}}(368\;K)$$

**Figure 9:**
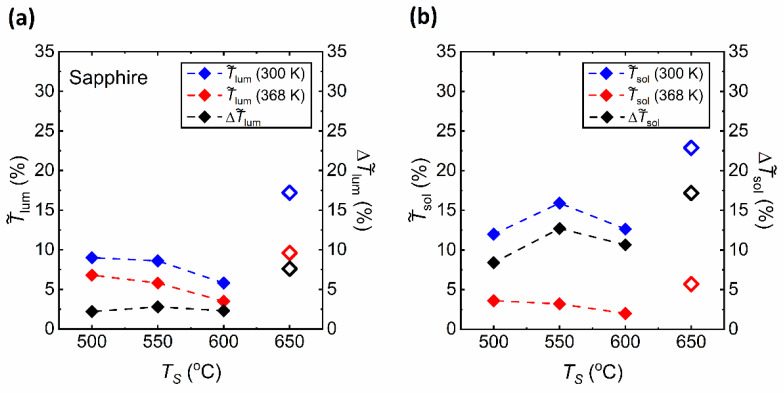
(**a**) Luminous transmittance (${\tilde{T}}_{\mathrm{lum}}$) at 300 K and 368 K and luminous modulation efficiency ($\Delta {\tilde{T}}_{\mathrm{lum}}$ ) obtained for films deposited at various substrates temperatures (*T_S_*). (**b**) Solar transmittance (${\tilde{T}}_{\mathrm{sol}}$ ) at 300 K and 368 K and solar modulation efficiency ($\Delta {\tilde{T}}_{\mathrm{sol}}$ ) obtained for films deposited at various substrates temperatures (*T_S_*). Data for the substrate temperature (*T_S_*) of 650 °C [17] are presented in hollow symbols.

The results of the calculations of ${\tilde{T}}_{\mathrm{lum},\mathrm{sol}}$ and $\Delta {\tilde{T}}_{\mathrm{lum},\mathrm{sol}}$ values are summarized in Table 4. Figure 9 gives the distribution of the data presented in Table 4. In Figure 9a, ${\tilde{T}}_{\mathrm{lum}}$ values obtained for VO_2_ thin films prepared at *T_S_* = 500, 550 and 600 °C in the insulating phase (300 K) appear to be smaller from that obtained for films prepared at *T_S_* = 650 °C. The ${\tilde{T}}_{\mathrm{lum}}$ values in the metallic phase (368 K) show a similar characteristic. Since the difference between ${\tilde{T}}_{\mathrm{lum}}$ values obtained at 300 and 368 K is not large due to the high optical transmittance between 450 and 700 nm, the $\Delta {\tilde{T}}_{\mathrm{lum}}$ values are small. Figure 9b presents ${\tilde{T}}_{\mathrm{sol}}$ and $\Delta {\tilde{T}}_{\mathrm{sol}}$ values of the as-prepared VO_2_ films which are also lower than those obtained for the film prepared at 650 °C [17]. However, the ${\tilde{T}}_{\mathrm{sol}}$ values at 368 K are indeed much lower compared to the ones at 300 K, due to the near-zero transmission in the NIR region integrated in Equation (2). This large difference between the ${\tilde{T}}_{\mathrm{sol}}$ values at 300 and 368 K is reflected on the large values of $\Delta {\tilde{T}}_{\mathrm{sol}}$.

### 3.4. Electrical MIT Characterization

**Figure 10:**
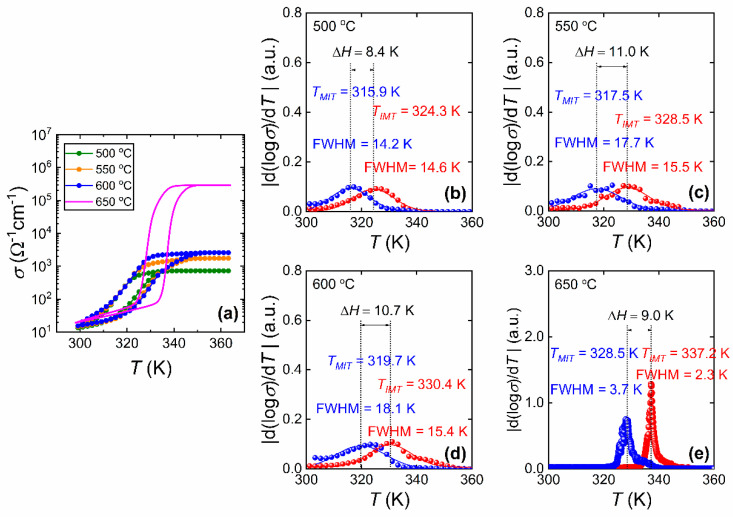
Temperature (*T*) dependence of electrical conductivity (*σ*) of VO_2_ films prepared at various substrate temperatures (*T_S_*). (**a**) Comparison of *T* dependence of *σ* of films prepared at *T_S_* = 500 °C, 550 °C, 600 °C, and 650 °C [32]. The phase transition characteristics for heating and cooling cycles for films prepared at *T_S_* = (**b**) 500 °C, (**c**) 550 °C, (**d**) 600 °C and (**e**) 650 °C [32].

**Figure 11:**
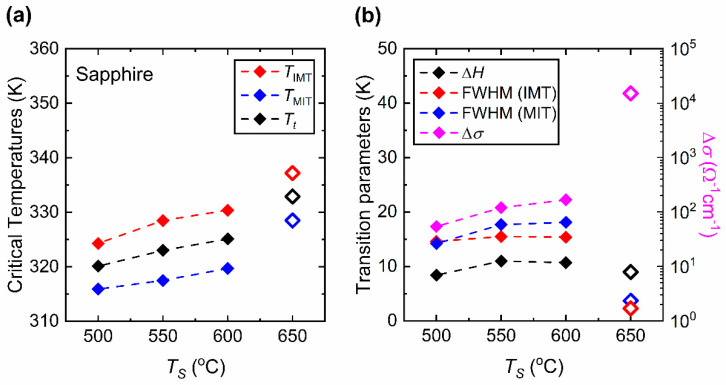
Electrical conductivity phase transition properties of VO_2_ films prepared on sapphire substrates at various substrate temperatures (*T_S_*). (**a**) Critical temperatures of the phase transition (*T*_IMT_, *T*_MIT_ and *T_t_*). (**b**) Phase transition parameters (Δ*H*, FWHM (IMT), and FWHM (MIT)) and the contrast of conductivity, Δ*σ* = *σ*(368 K)/*σ*(300 K). The data points for the substrate temperature 650 °C are from Ref. [32] and displayed in hollow symbols.

Figure 10a shows the temperature (*T*) dependence of the electrical conductivity (*σ*) of the VO_2_ films prepared at *T_S_* = 500, 550, 600 and 650 [32] °C, and their phase transition properties. The critical temperatures *T*_IMT_, *T*_MIT_ and the sharpness (FWHM) are obtained from the d(log(*σ*))/d*T* vs. *T*. plots as shown in Figure 10b–e. Here, it can be seen that there is only a slight broadening of the Δ*H*. On the other hand, the electrical phase transition properties of VO_2_ thin films studied in this work show a prominent difference compared to the film prepared at *T_S_* = 650 °C in Ref. [32]. The difference between the hysteresis properties of the samples is due to the difference in the nature of electrical and structural (observed optically) MIT [33,53]. Figure 11 gives the critical temperatures and phase transition parameters of VO_2_ films deposited on sapphire substrates obtained in *σ* vs. *T* measurements. As it is clear from the Figure 11a, the transition temperatures (*T*_IMT_, *T*_MIT_ and *T_t_*) drop with decreasing *T_S_*. In Figure 11b, the Δ*H* does not seem to be greatly affected from *T_S_*. As the *T_S_* is lowered from 650 to 550 °C, the Δ*H* broadens from 9 to 11 K, respectively. However, for *T_S_* = 500 °C, the Δ*H* drops to 8.4 K which shows narrower Δ*H* compared to the other VO_2_ films. On the other hand, the effect of *T_S_* appears to be prominent in the sharpness of both IM and MI transitions and the conductivity contrast (Δ*σ*). The FWHM of the films prepared between 500 and 600 °C are larger compared with those of the film prepared at 650 °C in both IM and MI transitions. The increase of FWHM, or loss of sharpness, in films between 500 and 600 °C indicates degradation of the abrupt phase transition. In addition, compared to the high-quality VO_2_ films prepared at 650 °C (Ref. [32]), the Δ*σ* of the films prepared at *T_S_* = 600 °C and below displays a substantial drop from the order of 4 to 2.

## 4. Discussion

**Figure 12:**
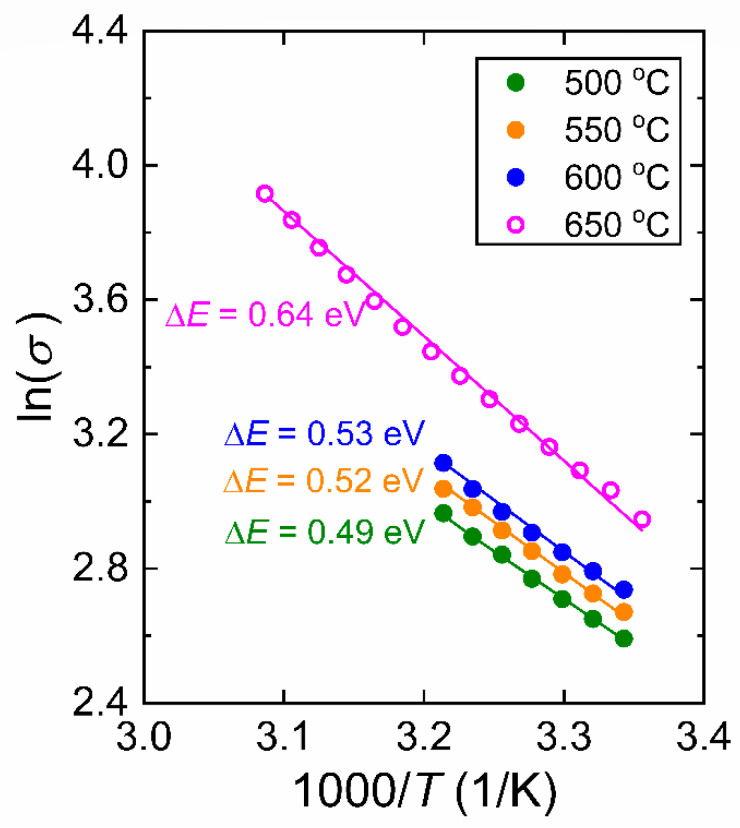
Arrhenius plot of conductivity (*σ*) data obtained from VO_2_ films in their insulating phases. Data set for VO_2_ film prepared at 650 °C is extracted from the *σ* vs. *T* plot in Ref. [32].

**Figure 13:**
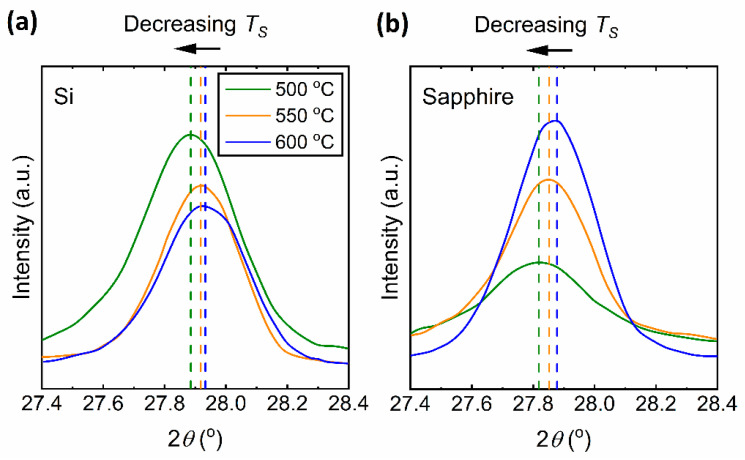
X-ray diffraction of (011) planes of VO_2_ thin films deposited at *T_S_* = 500, 550, and 600 °C on (**a**) Si and (**b**) sapphire substrates.

We begin our discussion by considering the structural investigation of VO_2_ thin films. The results presented in Figure 1, Figure 2, Figure 3 and Figure 4 clearly show that the *T_S_* has a considerable effect on the film structure, including both the crystallinity and surface roughness. For *T_S_* between 600 and 500 °C, both the XRD diffractograms and the Raman spectra show VO_2_ peaks, which disappear for *T_S_* ≤ 450 °C. From this observation, it can be understood that higher *T_S_* (≥500 °C) facilitates the formation of the VO_2_ phase, whereas lower temperature growth favors the formation of the V_2_O_5_ phase as can be referred to in Table 1. Since the V_2_O_5_ phase appears at lower *T_S_* and the root mean square roughness (*R_q_*) increases with lower *T_S_*, one may argue that there may be a correlation between the two observations. Nonetheless, we do not have any further experimental evidence to validate the latter. Yet, it is apparent from Figure 3 that low *T_S_* leads to a polycrystalline structure of misoriented grains with dissimilar geometries. Thus, not only the smaller grain size but also the increased defects in the films at low *T_S_* (e.g., 500 °C) may explain the drop in the $\tilde{T}$ spectrum. Secondly, the drop in the transition temperature (*T_t_*) can be related to the significance of unrelaxed lattice strain [54,55]. The degradation of the crystalline quality can be seen in the worsening of the phase transition properties given in Figure 7. Moreover, considering the electrical characterization, the degradation of contrast in the conductivity between the insulator and metallic phases (Δ*σ*) may be a result of the reduction of the current path due to increased oxygen vacancies and surface roughness [56]. We also examined the conductivity activation energy of the as-prepared VO_2_ films. The Arrhenius plot of ln(*σ*) vs. 1000/*T* is presented in Figure 12 where it is apparent that the conductivity (*σ*) of VO_2_ is thermally activated. We can represent the thermally activated behavior by,

(4) $$\sigma =\sigma_{o}\exp\left(\frac{\Delta E_{}}{2k_{B}T}\right)$$

where Δ*E* = *E_g_* if we assume the sample is intrinsic. Equation (4) yields Δ*E* of the VO_2_ films as 0.53 ± 0.02, 0.52 ± 0.01 and 0.49 ± 0.01 eV corresponding to prepared at *T_S_* = 600, 550 and 500 °C, respectively. The thermally activated conductivity can arise either from intrinsic conductivity (thermal generation across the bandgap) with Δ*E* = *E_g_* as in Equation (4) or from the ionization of defects or impurities in the film, in which case Δ*E* is not the bandgap but the magnitude of the energy difference between the transport band and the defect or impurity level. The Δ*E* values appear to be lower than that of high-quality VO_2_ films, which is 0.64 ± 0.01 eV from Ref. [36]. The Δ*E* values seem to follow a similar trend to that of Δ*σ* with respect to *T_S_* in Figure 11b. The drop in Δ*σ* and the apparent narrowing of Δ*E* may too be related to the increased density of grain boundaries and defects of oxygen vacancies due to low *T_S_* which is pointed out in Ref. [57]. In addition, Figure 13 presents the shifting of the amplified XRD peak of VO_2_ (011) at ~28° to a lower angle as *T_S_* decreases. This signifies an enlarged lattice parameter of VO_2_ due to defects and lattice strain, which is also suggested in Ref. [56]. It is worth comparing Δ*E* = 0.49–0.64 eV for these VO_2_ films with the indirect (smallest) optical bandgap reported for VO_2_ films deposited at 650 °C, which is 0.52 eV [17]. The work in Ref. [33] puts the optical bandgap between in the range 0.55–0.60 eV; again Δ*E* is very close to these values.

**Figure 14:**
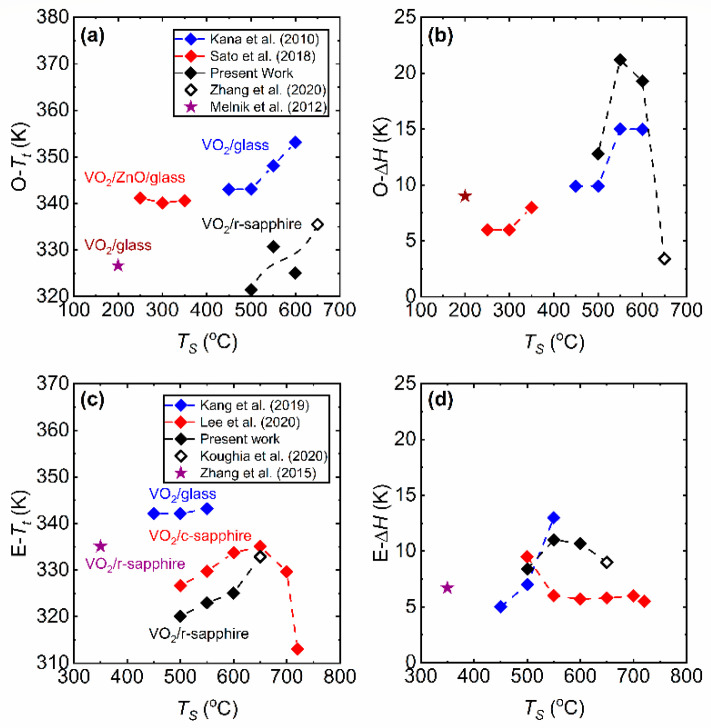
Comparison of VO_2_ thin films deposited at various temperatures studied in different works. (**a**) Comparison of *T_t_* obtained from optical (O-) switching measurements. (**b**) Hysteresis width (Δ*H*) of the films that are obtained from the temperature (*T*) dependence of optical transmittance ($\tilde{T}$). (**c**) Comparison of phase transition temperature (*T_t_*) obtained from electrical (E-) switching. (**d**) Hysteresis width (Δ*H*) of the films that are obtained from the *T*-dependence of electrical conductivity or resistance. Note that Kana et al. (2010) is [30]; Kang et al. (2019) is [57]; Koughia et al. (2020) is [32]; Lee et al. (2020) is [28]; Melnik et al. (2012) is [26]; Sato et al. (2012) is [21]; Zhang et al. (2015) is [36]; Zhang et al. (2020) is [17].

Finally, we compare our results with other works [21,28,30,57] in which the effect of *T_S_* on the phase transition properties of VO_2_ thin films have been studied. These studies have employed RF reactive inverted cylindrical magnetron sputtering [21], RF magnetron sputtering [28], inductively coupled plasma (ICP)-assisted sputtering [30], and pulsed-laser deposition (PLD) [57] for thin film preparation. We also consider selected works [26,36] which have reported the production of high-quality VO_2_ thin films at low substrate temperatures. Figure 14 presents the phase transition properties of VO_2_ thin films obtained by means of optical transmittance ($\tilde{T}$) and electrical conductivity (*σ*) measurements. Figure 14a,b show the comparison of the *T_t_* and Δ*H* characteristics of the films on sapphire substrates from the present work with the films prepared in Refs. [21,30]. It should be noted that the optical transmittance has been measured at different wavelengths. VO_2_ films reported in Ref. [26] were prepared at *T_S_* = 200 °C by reactive magnetron sputtering with a post-deposition annealing treatment at 300 °C, and the transition properties were obtained at *λ* = 2000 nm. The present work has used *λ* = 2500 nm, whereas *λ* = 1450 nm for Ref. [21] and 1100 nm for Ref. [30] were used. In Figure 14a, the films in Ref. [21] have a constant *T_t_* around 340 K, whereas the *T_t_* of films in Ref. [30] decreases from *Ts* = 600 to 500 °C and stays constant around 343 K until *Ts* = 450 °C. In contrast, the *T_t_* of the VO_2_ films on sapphire substrates presented in this work drops down to 323 K as the *T_S_* is lowered to 500 °C. As can be observed in Figure 14b, reducing the *T_S_* has a significant impact on the Δ*H* of the films prepared in the present work which can be related to the polycrystallinity as well as increased compressive and tensile strain in the films [28,32]. Figure 14c,d present the comparison of electrical phase transition properties obtained from Refs. [28,57]. Moreover, VO_2_ films reported in Ref. [36] were prepared by molecular beam epitaxy (MBE) at *T_S_* = 350 °C. In Figure 14c, the *T_t_* of the films of Ref. [28] increases from a low *T_S_* = 450 to 550 °C and then drops at high *T_S_* = 700 and 720 °C. On the contrary, in Figure 14d, the Δ*H* values appear to be relatively unaffected by *T_S_*, and they seem to be mostly constant around 5.5 K, only films prepared at 450 °C have a large Δ*H* around 10 K. The *T_t_* values obtained from Ref. [57] are constant around 343 K. In addition, the Δ*H* drops with decreasing *T_S_* from 13 K and 5 K. However, if the phase transition sharpness and Δ*σ* are taken into consideration, these films in Refs. [28,57] do not display attributes associated with high-quality films with decreasing *T_S_*. Overall, according to these comparisons, the preparation of high-quality VO_2_ films at low temperatures appear to be a challenge even though different deposition techniques and deposition parameters are applied. The degradation of the film properties may essentially rise from loss of crystalline quality, the degradation of stoichiometry (e.g., if there is an interface film between VO_2_ and the substrate) and the presence of defects and lattice strain that arise from film-substrate mismatch.

## 5. Conclusions

The effect of substrate temperature (*T_S_*) on the structural, optical and electrical properties of VO_2_ thin films during DC magnetron sputtering have been investigated. The results showed that the *T_S_* plays a crucial role on the microstructure and hence the optical and electrical phase transition properties of the VO_2_ films. It is found that *T_S_* below 500 °C favors the growth of the V_2_O_5_ phase where *T_S_* of 500 °C and higher facilitate the growth of stoichiometric VO_2_ films. The lowering of *T_S_* from 600 to 500 °C have shown reduced optical transmittance ($\tilde{T}$) and modulation efficiencies, broadened hysteresis width (Δ*H*), and decreased transition temperature, which suggests the presence of defects and unrelaxed lattice strain in the films. The comparison of the present results with those published on similar VO_2_ samples, where the effect of *T_S_* has been examined, indicates significant challenges remain in the fabrication of high-quality VO_2_ films below a *T_S_* of 650 °C.

## Figures and Tables

**Table 1:** Deposition conditions of VO_2_ thin films on Si and sapphire substrates.

Substrate Temperature (*T_S_*) (°C)	Oxygen Flow Rate (sccm)	Sputtering Power (W)	Pressure (Pa)	Duration (Hour)	Phase
350	1.3	100	1.33	2	V_2_O_5_
400	1.3	100	1.33	2	V_2_O_5_
450	1.3	100	1.33	2	V_2_O_5_
500	1.3	100	1.33	2	VO_2_
550	1.3	100	1.33	2	VO_2_
600	1.3	100	1.33	2	VO_2_
650 [17]	1.3	100	1.33	2	VO_2_

**Table 2:** Average grain size (GS_avg_), average (*R_a_*) and root-mean square (*R_q_*) roughness of VO_2_ thin films prepared at various substrate temperatures (*T_S_*).

Substrate Temperature (*T_S_*) (°C)	GS_avg_ (nm)	*R_a_* (nm)	*R_q_* (nm)	GS_avg_ (nm)	*R_a_* (nm)	*R_q_* (nm)
	Si			Sapphire		
500	104 ± 20	2	10	108 ± 16	4	8
550	116 ± 26	5	5	124 ± 27	2	6
600	145 ± 51	3	4	153 ± 59	2	3
650 [17]	114 ± 24	2	3	~Flat	2	2

**Table 3:** Optical transmittance ($\tilde{T}$ ) and transmittance contrast *(*$\Delta \tilde{T}$ ) values of VO_2_ films prepared at different substrate temperatures (*T_S_*) obtained at 300 K (insulating) and 368 K (metallic) for λ = 1500 nm, 2000 nm and 2500 nm.

Substrate Temperature (*T_S_*) (°C)	*λ* (nm)	${\tilde{T}}_{300K}$ (%)	${\tilde{T}}_{368K}$ (%)	$\Delta \tilde{T}$ (%)	${\tilde{T}}_{300K}$ (%)	${\tilde{T}}_{368K}$ (%)	$\Delta \tilde{T}$ (%)
			Si			Sapphire	
500	2500	34.5	0.5	34.0	35.0	0.6	34.4
	2000	27.6	0.5	27.1	33.3	0.7	32.6
	1500	16.9	0.5	16.4	24.4	0.7	23.5
550	2500	51.9	0.2	51.7	44.5	0.2	44.3
	2000	45.0	0.2	44.8	46.7	0.3	46.4
	1500	30.7	0.3	30.4	41.1	0.3	40.8
600	2500	48.2	0.1	48.1	43.7	0.1	43.6
	2000	42.0	0.1	41.9	48.7	0.1	48.6
	1500	27.3	0.1	27.2	31.8	0.2	31.6
650 [17]	2500	54.9	0.3	54.6	51.3	0.5	50.8
	2000	49.3	0.4	48.9	45.2	0.5	44.7
	1500	35.5	0.5	35.0	39.9	0.8	39.1

**Table 4:** Luminous and solar modulation efficiencies of VO_2_ films prepared on sapphire substrates at various *T_S_*.

Substrate Temperature (*T_S_*) (°C)	${\tilde{T}}_{\mathrm{lum}}(\%)$ (300 K)	${\tilde{T}}_{\mathrm{lum}}(\%)$ (368 K)	$\Delta {\tilde{T}}_{\mathrm{lum}}(\%)$	${\tilde{T}}_{\mathrm{sol}}(\%)$ (300 K)	${\tilde{T}}_{\mathrm{sol}}(\%)$ (368 K)	$\Delta {\tilde{T}}_{\mathrm{sol}}(\%)$
500	9	6.8	2.2	12	3.6	8.4
550	8.6	5.8	2.8	15.91	3.2	12.71
600	5.8	3.5	2.3	12.64	2	10.64
650 [17]	17.2	9.6	7.6	22.88	5.7	17.18

## Data Availability

Data is available from the corresponding author upon reasonable request.
